# Iguratimod improves bleomycin-induced pulmonary inflammation and fibrosis by regulating macrophage polarization through inhibiting the TLR4/NF-κB pathway

**DOI:** 10.3389/fimmu.2025.1558903

**Published:** 2025-03-13

**Authors:** Huan Xu, Kaixuan Ma, Ziting Ma, Tianyu Zhuang, Ling Lin

**Affiliations:** ^1^ Department of Rheumatology, First Affiliated Hospital of Shantou University Medical College, Shantou, China; ^2^ Laboratory of Molecular Cardiology, First Affiliated Hospital of Shantou University Medical College, Shantou, China; ^3^ Department of Gastroenterology, First Affiliated Hospital of Shantou University Medical College, Shantou, China; ^4^ Department of Endocrinology, First Affiliated Hospital of Shantou University Medical College, Shantou, China; ^5^ Department of Rheumatology, Shantou University Medical College, Shantou, China

**Keywords:** interstitial pulmonary fibrosis, inflammation, iguratimod, M1 polarization, TLR4

## Abstract

**Introduction:**

Pulmonary fibrosis (PF) is a fatal pathological subtype of interstitial lung disease, frequently manifests as a pulmonary complication of connective tissue disease. Iguratimod (IGU) is a new class of anti-rheumatic drugs used in the treatment of rheumatoid arthritis (RA). Studies have reported that RA patients treated with IGU have better lung function, and IGU effectively ameliorates PF. However, the mechanism by which IGU improves PF is still unclear. This study aims to elucidate the therapeutic efficacy and mechanisms of IGU in PF through in vivo and in vitro investigations, so as to provide a new treatment method for PF.

**Methods:**

In our research, bleomycin (BLM)-induced PF of mice were used to observe the therapeutic effect of different concentrations of IGU. And the effects of IGU on macrophage polarization and activation pathway TLR4/NF-κB in lung tissue were analyzed. In addition, Raw264.7 macrophages were induced to M1 and M2 polarization in vitro, and the effects of IGU on Raw264.7 macrophage polarization and related pathways were observed.

**Results:**

In our study, database analysis suggested that macrophage polarization-relative genes and pathways as well as TLR4 activation played important roles in BLM-induced PF in mice. Besides, we found that IGU effectively ameliorated BLM-induced PF and epithelial-mesenchymal transition in mice, and inhibited the polarization of M1/M2 macrophages at different stages of PF. Moreover, In vitro studies further demonstrated that IGU suppressed M1 polarization of Raw264.7 and its activation pathway TLR4/NF-κB.

**Discussion:**

In summary, IGU inhibits the activation of macrophages and M1 polarization through inhibiting the TLR4/NF-κB pathway, thereby improving BLM-induced pulmonary inflammation and fibrosis in mice. It is suggested that IGU may be a new therapeutic option for interstitial pulmonary fibrosis.

## 1 Introduction

Interstitial lung disease (ILD), a diverse group of diffuse lung disorders that predominantly affect the lung interstitium and alveolar spaces, is characterized by alveolar inflammation and interstitial fibrosis as the fundamental pathological changes, leading to the loss of the alveolar-capillary functional units (1). Depending on the etiology, pathological characteristics and clinical manifestations, ILD can be further classified into various types, such as nonspecific interstitial pneumonia (NSIP), idiopathic pulmonary fibrosis (IPF) and connective tissue disease-associated ILD (CTD-ILD) (2). Despite variations in their underlying causes and mechanisms, ILD often progresses to pulmonary fibrosis (PF), which profoundly impacts patient prognosis and quality of life (3). Nintedanib, pirfenidone, glucocorticoids and immunosuppressants are used in the treatment of PF in clinical practice, but are accompanied with some adverse reactions and drug tolerance (4, 5). Therefore, the research and development of new drugs and new treatment methods are particularly urgent.

PF is a common pathological type of advanced stage ILD. Emerging evidence highlights the pivotal role of macrophage polarization in the progression of PF (6). Macrophages are more prone to M1 polarization and secrete a large amount of interleukins and tumor necrosis factor in the early stage of fibrosis, which promote alveolar inflammation (7). Due to long-term inflammatory stimulation, macrophages begin to shift toward M2-like polarization and secrete TGF-β and other factors. This phenotypic shift contributes to aberrant tissue repair, facilitating epithelial-mesenchymal transition (EMT) in alveolar epithelial cells and promoting the development of myofibroblast foci. Excessive secretion of extracellular matrix (ECM) induces the formation of fibrous scars and honeycomb lung, and eventually leads to the destruction of lung structure and loss of function (8).

Iguratimod (IGU) is currently mainly used in China and Japan for the treatment of rheumatoid arthritis (RA), which is initially developed as a cyclooxygenase inhibitor. As a conventional synthetic disease-modifying anti-rheumatic drug, IGU has a good safety profile and displays fewer side effects (9, 10). At present, the specific pharmacological mechanism of IGU is still unclear. According to prior studies, IGU effectively inhibits the release of prostaglandins from fibroblasts *in vitro*, similar to selective COX-2 inhibitors (11). In addition, IGU can inhibit the production of immunoglobulin in B cells (12), and inhibit the production of TNF-α and other inflammatory factors, thereby exerting immunomodulatory and anti-inflammatory functions (13). Furthermore, IGU has the effect of inhibiting bone resorption and promoting bone formation (14). In recent years, the clinical application of IGU has been expanded and is being widely explored in other rheumatic diseases, such as ankylosing spondylitis (15), primary Sjogren’s syndrome (16), systemic sclerosis (17) and IgG4-related disease (18). In addition, studies have reported that IGU has an anti-PF effect (19), but the specific mechanism of action is still unclear. In the present study, *in vivo* and *in vitro* experiments were undertaken to investigate the impact of IGU on macrophage polarization, and to explore whether IGU could improve PF by regulating macrophage polarization, so as to further elucidate the pharmacological effect of IGU.

## 2 Materials and methods

### 2.1 Reagents and antibodies

Bleomycin (BLM) was purchased from Nippon Kayaku Co., Ltd. (Tokyo, Japan). IGU was purchased from Aladdin (#I124859, China). Dimethyl sulfoxide (DMSO), Sodium carboxymethyl cellulose (CMC-Na) and lipopolysaccharide (LPS) were purchased from Solarbio (Beijing, China). Mouse IL-4 and mouse IL-13 were purchased from PeproTech (Rocky Hill, USA). Antibodies anti-TLR4 (#NB100-56566SS), and anti-CD86 (#NBP2-25208) were purchased from Novus. Anti-β-actin (#4970S), anti-Arg-1 (#93668), anti-CD206 (#24595), anti-α-smooth muscle actin (α-SMA) (#19245), anti-vimentin (#5741) and anti-E-cadherin (#3195)were purchased from Cell Signaling Technology (Boston, USA). Anti-iNOS (#ab178945), anti-NF-κB (#ab32536), anti-phosphorylated NF-κB (#ab131100) and anti-collagen I (#ab34710) were purchased from Abcam (UK).

### 2.2 Animals and treatments

Forty C57BL/6J mice (approximately 20g, 6–8 weeks old) were obtained from Zhuhai BesTest Bio-tech Co., Ltd. (Zhuhai, China). All mice were maintained at 22°C, 50% humidity, specific pathogen-free ambient conditions, and a 12-hour light/dark cycle. The animals were randomly allocated into four groups: (1) Control, (2) BLM, (3) BLM+IGU 25 mg/kg, and (4) BLM+IGU 50 mg/kg. Groups 2-4 were injected intratracheally with 3 mg/kg BLM on day 0, while the control group was injected with an equivalent volume of saline. Thereafter, IGU, dissolved in 0.4% CMC-Na, was administered daily by intragastric administration. Groups 1 and 2 were given an equivalent amount of solvent as placebo control. Five mice in each group were euthanized in the alveolar inflammatory stage on day 7 and in the pulmonary fibrotic stage on day 28. At the end of the animal experiment, the mice were anesthetized with 1% sodium pentobarbital (50mg/kg). Bronchoalveolar lavage fluid (BALF) and lung tissue were collected for subsequent experiments. After the operation, the animals were sacrificed by cervical dislocation.

### 2.3 Histopathology

The left lung tissues of mice were fixed in 4% paraformaldehyde for 6 hours, followed by an 8-hour rinse in running water. After dehydration, lung tissue was made into wax blocks and 4μm-thick paraffin sections for subsequent experiments. Paraffin sections were deparaffinized with xylene and rehydrated through a graded alcohol seriesl. HE staining was performed with hematoxylin and eosin, and the Szapiel’s score was used to evaluate inflammation of lung tissue. Masson staining was performed using the corresponding kit according to the instructions (Solarbio, Beijing, China), and pulmonary fibrosis was assessed using the Ashcroft score (20).

### 2.4 Immunohistochemistry and immunofluorescence

Immunohistochemistry and immunofluorescence were used to detect the localization and characterization of related proteins in lung tissue of mice. After deparaffinization with xylene and hydration with gradient alcohol, paraffin sections were antigen repaired in sodium citrate at 100°C for 30 minutes, followed by complete removal of peroxide and blocking, according to the immunohistochemical kit (MXB, China) instructions. Subsequent paraffin sections were incubated overnight at 4°C using anti-collagen I (ab34710, 1:100). On the following day, after incubation with secondary antibodies, protein visualization was achieved using DAB staining, and paraffin sections were counterstained with hematoxylin. For immunofluorescence, after blocking with 5% BSA for 1 hour, the paraffin sections were incubated overnight at 4°C with anti-rabbit-CD206 (1:100, CST) and anti-mouse-CD86 (1:20, Novus) antibody. The second day, after incubation with Alexa Fluor 488-conjugated anti-rabbit IgG H&L (ab150077, Abcam) and Alexa Fluor-conjugated anti-mouse IgG H&L (ab150116, Abcam) at room temperature for 1 hour. Finally, the sections were mounted using an anti-fade mounting medium containing DAPI for nuclear staining.

### 2.5 Detection of hydroxyproline content

HYP content in lung tissue reflects the metabolism of collagen and degree of fibrosis (21). Detection methods were performed according to the corresponding instruction of HYP kit (BC0255, Solarbio, Beijing, China). 0.02g of the right upper lung of each mouse was collected for the determination of HYP content. After dissolving and carbonizing the tissues using 6 mol/L hydrochloric acid extract, the pH was adjusted to 6-8 using NaOH. The sample was hydrolyzed to produce free HYP, which was further oxidized by chloramine T. The oxidation product reacts with p-dimethylaminobenzaldehyde to produce a red compound, which has a characteristic absorption peak at 560 nm. The content of HYP can be calculated by measuring the absorbance at 560 nm of the hydrolyzed sample solution.

### 2.6 Proteins expression levels determination using Western blot

Proteins were extracted from lung tissues of mice and Raw264.7 cells using RIPA lysis buffer in order to detect the relative expression levels of target genes. 20 μg per sample of protein were subjected to polyacrylamide gel electrophoresis and subsequently transferred onto polyvinylidene difluoride (PVDF) membranes. The membranes were blocked with 5% skim milk powder for one hour at room temperature. Next, the membranes were incubated overnight at 4°C using the anti-mouse CD86 (1:200), anti-rabbit iNOS (1:1000), anti-rabbit CD206 (1:1000), anti-rabbit Arg-1 (1:800), anti-rabbit E-cadherin (1:1000), anti-rabbit vimentin (1:1000), anti-rabbit α-SMA (1:1000), anti-rabbit NF-κB (1:1000), anti-rabbit phosphorylated NF-κB (1:500) and anti- mouse TLR4 (1:1000). The following day, membranes were washed three times with Tris-buffered saline containing 0.1% Tween-20 (TBST) for 10 minutes per wash and subsequently incubated with secondary antibodies: HRP-conjugated anti-rabbit IgG (CST, 1:3500) or HRP-conjugated anti-mouse IgG (Proteintech, 1:5000) for one hour at room temperature. Chemiluminescence detection was performed, and β-actin (CST, 1:1000) served as the internal control for western blot analysis.

### 2.7 Cytokine determination using Enzyme-linked immunosorbent assay

BALF of mice and cell culture supernatants of Raw264.7 cells were collected to detect the levels of related cytokines, including TNF-α, IL-6, IL-1β and TGF-β. Detection methods were performed according to the corresponding instruction of ELISA kit (MEIMIAN, Jiangsu, China).

### 2.8 Cell culture and treatments

Raw264.7 cells were obtained from the Cell Bank of the Chinese Academy of Sciences (Shanghai, China), and type II alveolar epithelial cells were sourced from Procell (Wuhan, China). The cells were cultured separately in DMEM and F-12K media (YEASEN, Shanghai, China), each supplemented with 10% fetal bovine serum (FBS, YEASEN, Shanghai, China). All cultures were maintained at 37°C in a humidified atmosphere with 5% CO_2_. M1 macrophage polarization was induced by treatment with lipopolysaccharide (LPS, 100 ng/mL), while M2 macrophage polarization was achieved by exposure to interleukin-4 (IL-4, 20 ng/mL) and interleukin-13 (IL-13, 20 ng/mL) following 48 hours of serum starvation. IGU was dissolved in DMSO and prepared as working solutions of 25 μg/mL and 50 μg/mL in DMEM for treatment. The control group received treatment with the corresponding solvent.

### 2.9 RNA expression levels determination using quantitative real-time polymerase chain reaction analysis

QRT-PCR was used to detect the RNA expression levels of related genes. RNA was extracted from Raw264.7 cells by Trizol. 500ng RNA was converted into complementary DNA according to the reverse transcription kit (#11141ES60, YEASEN, China), followed by qRT-PCR experiments using the SYBR Green kit (#11185ES03, YEASEN, China). β-actin was used as an internal reference, and Ct method (2^-ΔΔCt^) was used to calculated the relative expression levels of genes. Primer sequences are presented in **Table 1**.

**Table 1:** Primer sequences of genes for quantitative real time-PCR.

Gene	Forward primer	Reverse primer
TNF-α	GCCCATGTTGTAGCAAACCC	GGAGGTTGACCTTGGTCTGG
IL-6	GACAAAGCCAGAGTCCTTCAGA	TGTGACTCCAGCTTATCTCTTGG
IL-1β	TGCCACCTTTTGACAGTGATG	TGATGTGCTGCTGCGAGATT
MMP2	GCCCCCATGAAGCCTTGTTT	TAGCGGTCTCGGGACAGAAT
MMP9	CAGCCGACTTTTGTGGTCTTC	CGGTACAAGTATGCCTCTGCCA
β-actin	GGCGGACTGTTACTGAGCTG	CTGCGCAAGTTAGGTTTTGTCA

### 2.10 Cell proliferation and IGU toxicity assessment using Cell counting Kit−8 assay

CCK-8 assay was employed to evaluate the proliferation of RAW264.7 cells and assess the cytotoxicity of IGU. After the *in vitro* model of 96-well plate was completed, 10μl CCK8 and 90μL medium were added into each well and incubated for 30 minutes. Absorbance at 450 nm was then measured using a microplate reader to determine cell viability and analyze the results.

### 2.11 Detection of cellular protein markers using flow cytometry

Following PBS rinsing, RAW264.7 cells were blocked with 10% goat serum at room temperature for 1 hour. The cells were then incubated with anti-CD86 (APC, 1:100, BioLegend) and anti-F4/80 (FITC, 1:100, BioLegend) antibodies for 15 minutes at room temperature. After washing with PBS, the cells were permeabilized with 0.5% Triton X-100 for 10 minutes. Subsequently, the cells were rinsed again with PBS and incubated with anti-CD206 (PE, 1:80, BioLegend) for 15 minutes at room temperature. Finally, the prepared cell suspension was filtered and analyzed using flow cytometry.

### 2.12 Detection of Matrix metalloproteinases levels using gelatin zymography assay

MMPs, such as MMP2 and MMP9, in the supernatants of Raw264.7 cells were characterized by gelatin zymography where 20 μg protein of cell supernatant from each group was subjected to sodium dodecyl sulfate-polyacrylamide gel electrophoresis (SDS-PAGE) containing gelatin (22). At the end of the electrophoresis, the gel was washed twice with eluent for 30 minutes each to remove SDS from the gel. Subsequently, the gels were incubated at room temperature for 24 h to allow MMPs to break down the gelatin. Then, the bands were stained and destained for 1 h on a shaker until clear.

### 2.13 Cell transfection

Overexpression of TLR4 (NM_021297.3) shRNA and negative control (NC) - shRNA were gained from Obio Technology Corp., Ltd.(Shanghai, China). The GL180 plasmid was used as the vector. Raw264.7 cells were transduced with shRNA-encoding lentivirus with 80 pfu/cell. Then, transfection efficiency was assessed by western blot.

### 2.14 Data collection and processing for bioinformatics

“Pulmonary Fibrosis” and “Bleomycin” were used as a qualifier for retrieval In the GEO database, the GSE40151 dataset was selected for analysis. In the “affy” package of R language, the differentially-expressed genes between the pulmonary fibrosis group and the control group at different stages according to an FDR<0.05 and |log2FC|>1 through the “limma” package. The “ggplot2” package was used to draw volcano plots. The related target pathway genes were visualized as heat maps using the “pheatmap R” package.

### 2.15 KEGG and GO enrichment analysis

“Enrich KEGG” and “enrich GO” functions of the “cluster profiler” package in R language were used to perform KEGG and GO analysis of the screened differential genes. The “ggplot2” and “ggpubr” packages were used to draw bar charts and bubble charts, respectively.

### 2.16 Assessment of the immune microenvironment

A signature TIICs matrix “LM22” with 1000 permutations was set using the “CIBERSORT” package to calculate the infiltration fraction of lymphocytes categorize into 22 different cell types, whose constituent ratios sum to one. To evaluate the infiltration of immune cells between mice with pulmonary fibrosis and normal controls. Finally, the Wilcoxon test was employed to evaluate the statistical significance of differences in immune cell infiltration between the two groups.

### 2.17 Statistical analysis

GraphPad Prism 8 and SPSS 23.0 software were used to analyzed all data. For datasets with homogeneous variance and normal distribution, one-way *ANOVA* followed by the least significant difference (*LSD*) *t*-test was applied, with results expressed as mean ± standard deviation (mean ± SD). In cases of unequal variance, the *F*-test was used instead, followed by *Dunnett’s T3* test. Nonparametric tests were used for datasets that did not meet the criteria for normal distribution. A *p*-value < 0.05 were considered as statistically significant.

## 3 Results

### 3.1 IGU ameliorates BLM-induced pulmonary inflammation and fibrosis *in vivo*

**Figure 1:**
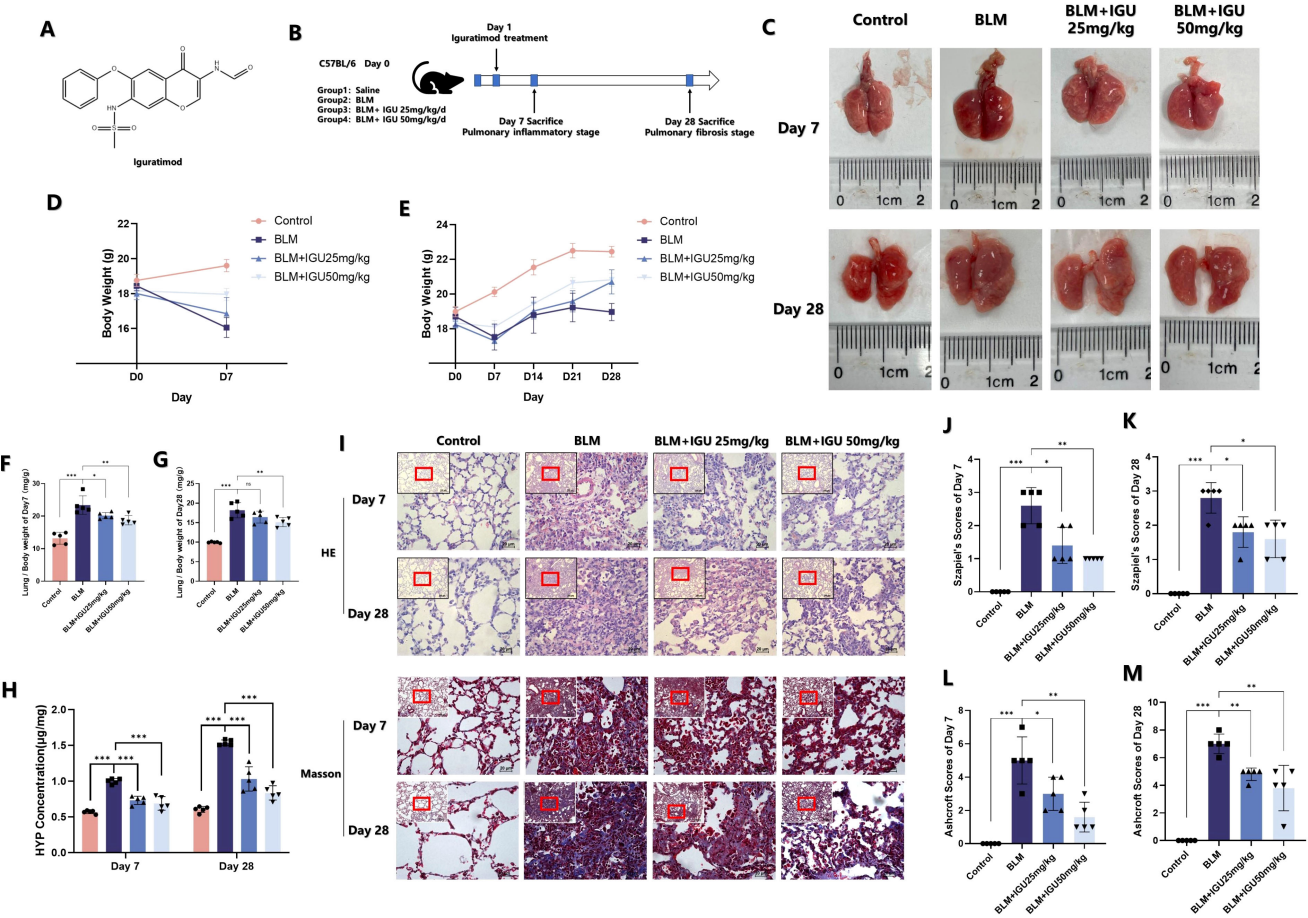
IGU reduces bleomycin-induced pulmonary inflammation and fibrosis *in vivo*. **(A)** Molecular structure of IGU. **(B)** Schematic diagram of IGU intervention in the *in vitro* bleomycin-induced PF model. Changes in lung tissue **(C)**, body weight **(D, E)** and lung weight index **(F, G)** of mice in each group after IGU intervention. **(H)** HYP content in the lungs of mice in each group after IGU intervention. **(I–M)** HE staining and corresponding Szapiel score, Masson staining and corresponding Ashcroft score of lung tissue in each group of mice. (n=5). *P<0.05, **P<0.01, ***P<0.001, ns, No significance.

IGU, a nonsteroidal anti-inflammatory drug (NSAID), is a compound composed of a sulfonyl group, an aldehyde group, an imine group, a carbonyl group and an ester group (**Figure 1A**). Mice were given IGU by gavage 24 hours after intratracheal injection of BLM to induce PF, and were euthanized at the inflammatory stage on the 7th day and at the fibrotic stage on the 28th day to observe the effect of IGU treatment (**Figure 1B**). After intratracheally injected BLM, the volume and the weight of lung tissue were increased due to fibrosis, and the mice gained weight slowly. After treatment with 25 mg/kg or 50 mg/kg IGU, the above symptoms were improved (**Figures 1C–E**). As shown in **Figures 1F, G**, lung weight indices in mice with PF were increased (lung weight/body weight) due to inflammatory edema, collagen deposition in lung tissue and decreased body weight, which were also decreased after 7 and 28 days of IGU treatment (*p*<0.05).

Hydroxyproline (HYP) is one of the main components of collagen, which is mostly distributed in skin, tendon, cartilage and blood vessels. Therefore, the detection of HYP content in lung tissue can reflect the metabolism of collagen and degree of fibrosis (23). Lung inflammation and fibrosis of mice were jointly evaluated by combining the scores of HE staining and Masson’s trichrome staining. Seven days after BLM-induced PF, lung tissue of mice showed more inflammatory cell infiltration, and the alveolar wall became thickened and swollen, and the alveolar structure became disordered, with fibrotic lesions and collagen deposition visible. Szapiel’s scores and Ashcroft scores, as well as HYP content, were all higher than those in the control group. However, as shown in **Figure 1H–M**, this phenomenon could be reversed 25 mg/kg or 50 mg/kg IGU treatment (*p*<0.05).

### 3.2 IGU inhibits BLM-induced EMT *in vivo*

**Figure 2:**
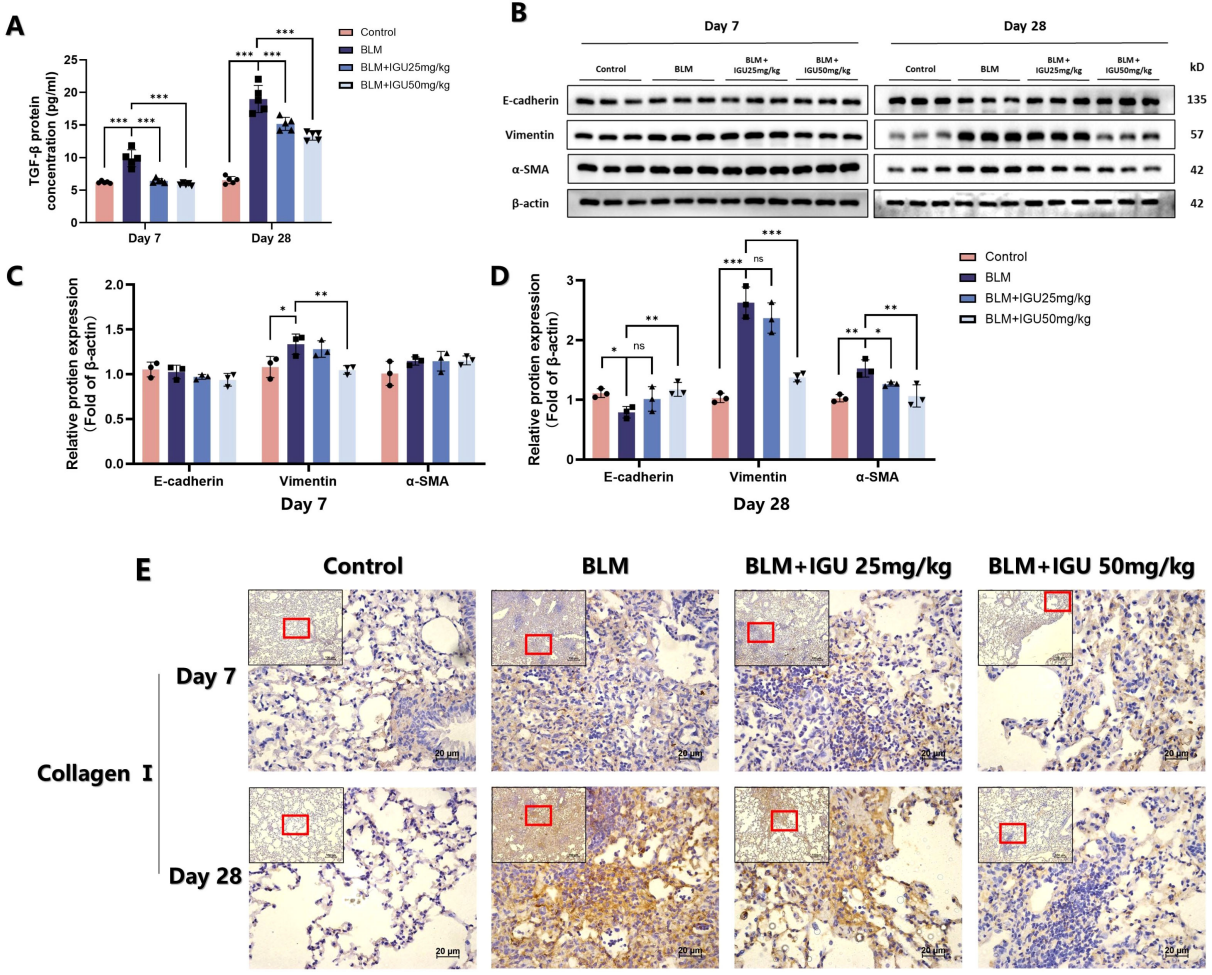
IGU inhibits bleomycin-induced EMT *in vivo*. **(A)** Content of TGF-β in bronchoalveolar lavage fluid of mice in each group treated with iguratimod after bleomycin-induced pulmonary fibrosis (*n*=5 per group). **(B–D)** Protein levels of E-cadherin, vimentin and α-SMA in lung tissue of mice in each group, β-actin was used as an internal control (*n*=3 per group). **(E)** Immunohistochemistry was used to determine the expression of collagen I in lung tissue of mice (n=5). *P<0.05, **P<0.01, ***P<0.001, ns, No significance.

EMT is an important phenotypic change in PF. Markers related to EMT were characterized in order to explore the possible mechanism of IGU in improving PF. The content of TGF-β in BALF, secreted by immune cells such as macrophages, which could promote the transformation of alveolar epithelial cells into mesenchymal cells (24), was quantified by ELISA on days 7 and 28 after BLM-induced PF. Treatment with different concentrations of IGU ameliorated BLM-induced TGF-β elevation (*p*<0.001, **Figure 2A**). In addition, protein expression levels of epithelial marker E-cadherin, and mesenchymal markers vimentin and α-SMA in lung tissues were detected via western blotting. Expression levels of EMT-related proteins, except for vimentin, did not change after 7 days of BLM induction. However, by day 28, BLM induction resulted in decreased E-cadherin expression, accompanied by increased levels of vimentin and α-SMA. Notably, treatment with IGU reversed these changes in a dose-dependent manner (*p*<0.05, **Figures 2B–D**). Immunohistochemical analysis revealed that 7 days after BLM-induced PF, a small amount of collagen I deposition and disrupted tissue structure were evident in the lungs of the BLM group. These changes were attenuated in the BLM+IGU25 and BLM+IGU50 treatment groups. By day 28, extensive fibrotic lesions and significant collagen I deposition were observed in the BLM group, whereas these pathological changes were markedly reduced in the BLM+IGU25 and BLM+IGU50 groups (**Figure 2E**).

### 3.3 IGU inhibits macrophage polarization in BLM-induced PF

**Figure 3:**
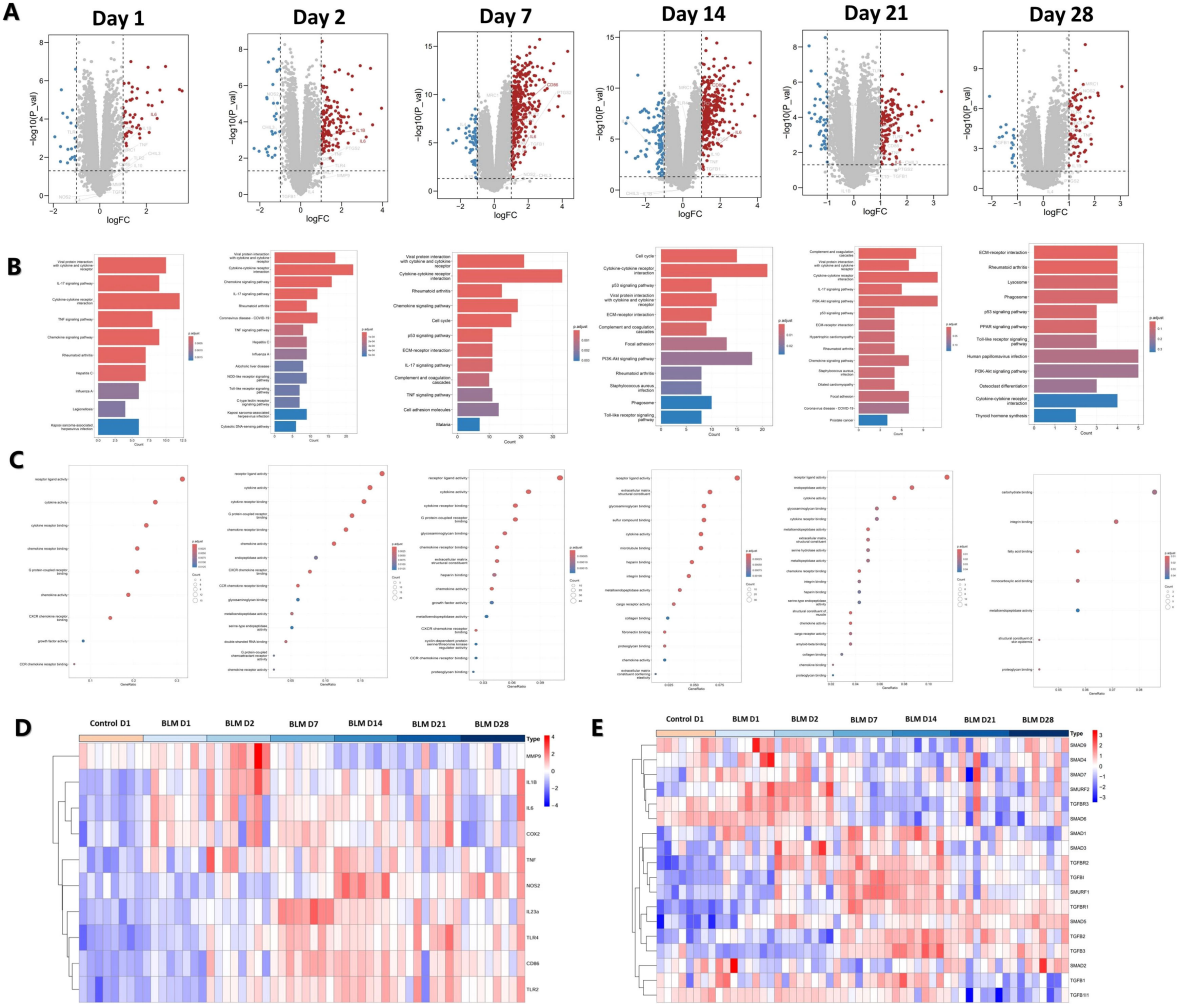
Analysis of macrophage polarization at different stages of BLM-induced PF in mice. **(A)** Volcano map of differentially-expressed genes. **(B)** KEGG and **(C)** GO functional enrichment analysis of differentially-expressed genes. **(D)** Heat map of M1 macrophage polarization-related gene expression. **(E)** Heat map of TGF-β pathway gene expression associated with M2 macrophage polarization.

Macrophages play a pivotal role in BLM-induced PF, exhibiting different types at different stages of PF. GSE40151 dataset analysis showed that M1 macrophage-associated genes, such as CD86, IL-1β and IL-6 were increased in BLM mice during the inflammatory phase within 14 days (**Figure 3A**). KEGG and GO enrichment analyses indicated that differentially expressed genes in this phase were predominantly involved in pathways such as the “IL-17 signaling pathway”, “Cytokine and cytokine receptor interaction”, “TNF signaling pathway”, “receptor ligand activity”, “cytokine activity”, “cytokine receptor binding” and other signaling pathways related to inflammation. In addition. In the PF stage, after 14 days of BLM induction, KEGG and GO enrichment analyses demonstrated that differentially expressed genes were increasingly associated with pathways related to cell proliferation, tissue repair, and extracellular matrix formation, including “Cell cycle”, “ECM receptor interaction”, “extracellular matrix structural components” (**Figures 3B, C**). Heat map visualization further highlighted the temporal dynamics of gene expression. M1 macrophage polarization-related genes, such as IL-1β, TNF and IL-6, showed an early increase on days 1-2 post-induction, with most markers, including CD86 and iNOS, peaking during days 7-4 (**Figure 3D**). M2 macrophages can secrete a large amount of TGF-β and other anti-inflammatory cytokines, and activate the TGF-β signaling pathway to promote pulmonary fibrosis. The expression levels of TGFβR1, TGFβR2, TGF-β1, TGF-β2, TGF-β3, Smad2 and Smad3 gradually increased from the 7th to the 28th day of BLM induction, while the expression levels of Smad6 and Smad7 decreased (**Figure 3E**). These results suggest that there are different dominant polarization types of macrophages at different disease stages of BLM-induced PF in mice. M1 macrophages were the dominant macrophages in the inflammatory stage, while M2 macrophages were the dominant macrophages in the fibrotic stage.

**Figure 4:**
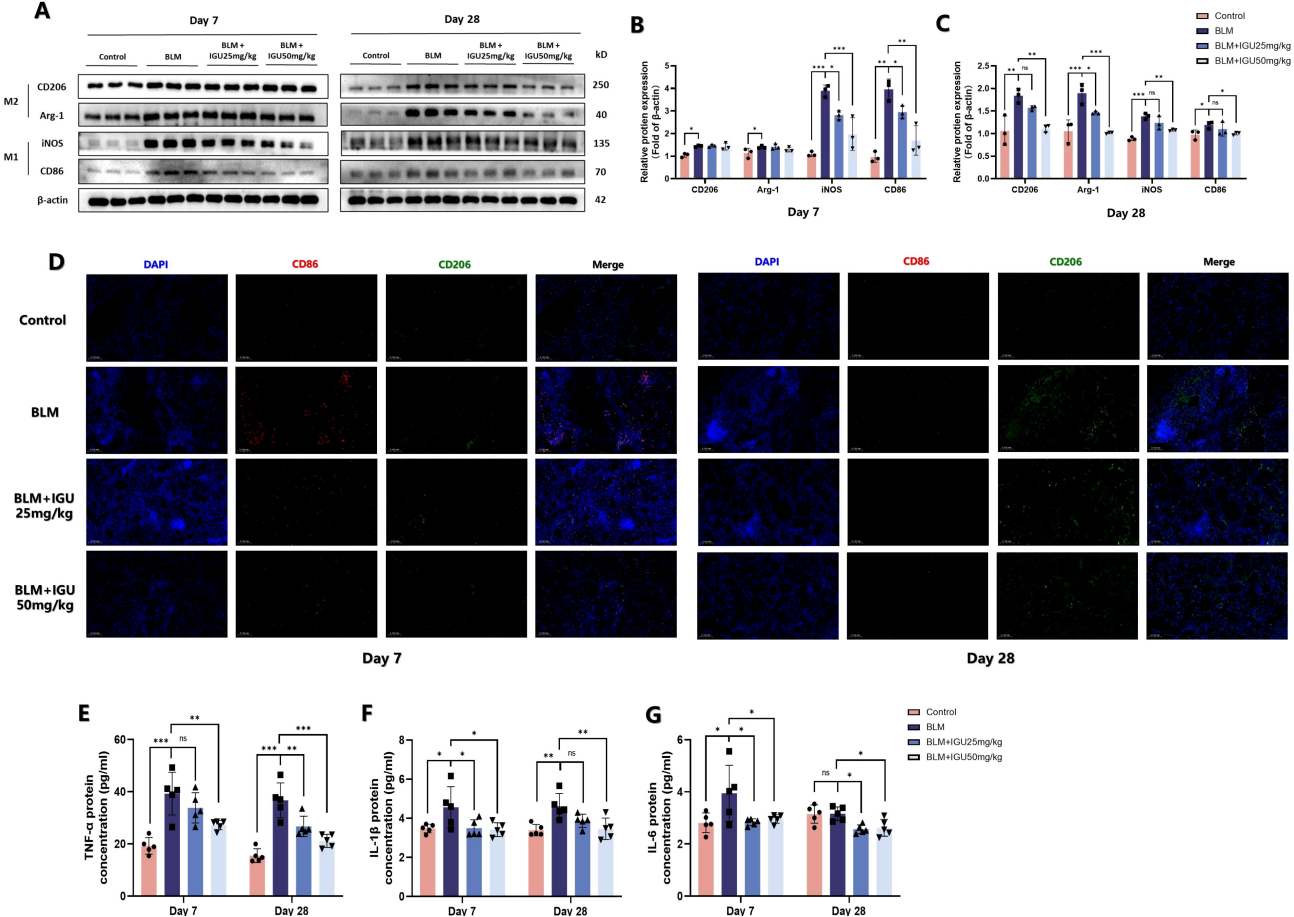
IGU inhibits macrophage polarization in BLM-induced PF *in vivo*. Representative western blots **(A)**, western blot quantitation **(B, C)**, and immunofluorescence **(D)** for the protein levels of M1 macrophage-related genes CD86 and iNOS, and M2 macrophage-related genes CD206 and Arg-1 in lung tissues of mice at different stages of BLM-induced PF, β-actin was used as an internal control (*n*=3). **(E–G)** Levels of M1 macrophage-associated inflammatory cytokines TNF-α, IL-6 and IL-1β in bronchoalveolar lavage fluid were quantified by ELISA (n=5). *P<0.05, **P<0.01, ***P<0.001, ns, No significance.

Immunofluorescence staining of CD86- and CD206-positive macrophages was conducted to elucidate the relationship between IGU treatment and macrophage polarization in BLM-induced PF. Following 7 days of BLM administration, CD86-positive macrophages in lung tissue of BLM mice were increased, but were reduced in BLM+IGU25 and BLM+IGU50 mice in a dose-dependent manner. The number of CD206-positive macrophages, which predominated after 28 days of BLM treatment, was also reduced by IGU treatment (**Figure 4D**). Western blot analysis further supported these findings, showing that the protein expression levels of M1 macrophage markers, CD86 and iNOS, and M2 macrophage markers, CD206 and Arg-1, were modulated in accordance with IGU treatment (*p*<0.05, **Figures 4A–C**). Additionally, ELISA measurements revealed the cytokine profiles associated with macrophage polarization in BALF. Levels of pro-inflammatory cytokines, including TNF-α, IL-1β, and IL-6, were significantly elevated in BALF of mice with BLM-induced PF at both 7 days and 28 days post-treatment. However, these cytokine levels were substantially reduced in the IGU-treated groups compared to the BLM group (*p*<0.05, **Figures 4E–G**). In short, these results highlight the presence of distinct macrophage polarization states during different stages of BLM-induced PF. Moreover, IGU effectively mitigates macrophage polarization in lung tissues, suggesting its potential therapeutic role in modulating macrophage-driven PF.

### 3.4 IGU alleviates type II alveolar epithelial cell inflammation caused by M1 macrophages

**Figure 5:**
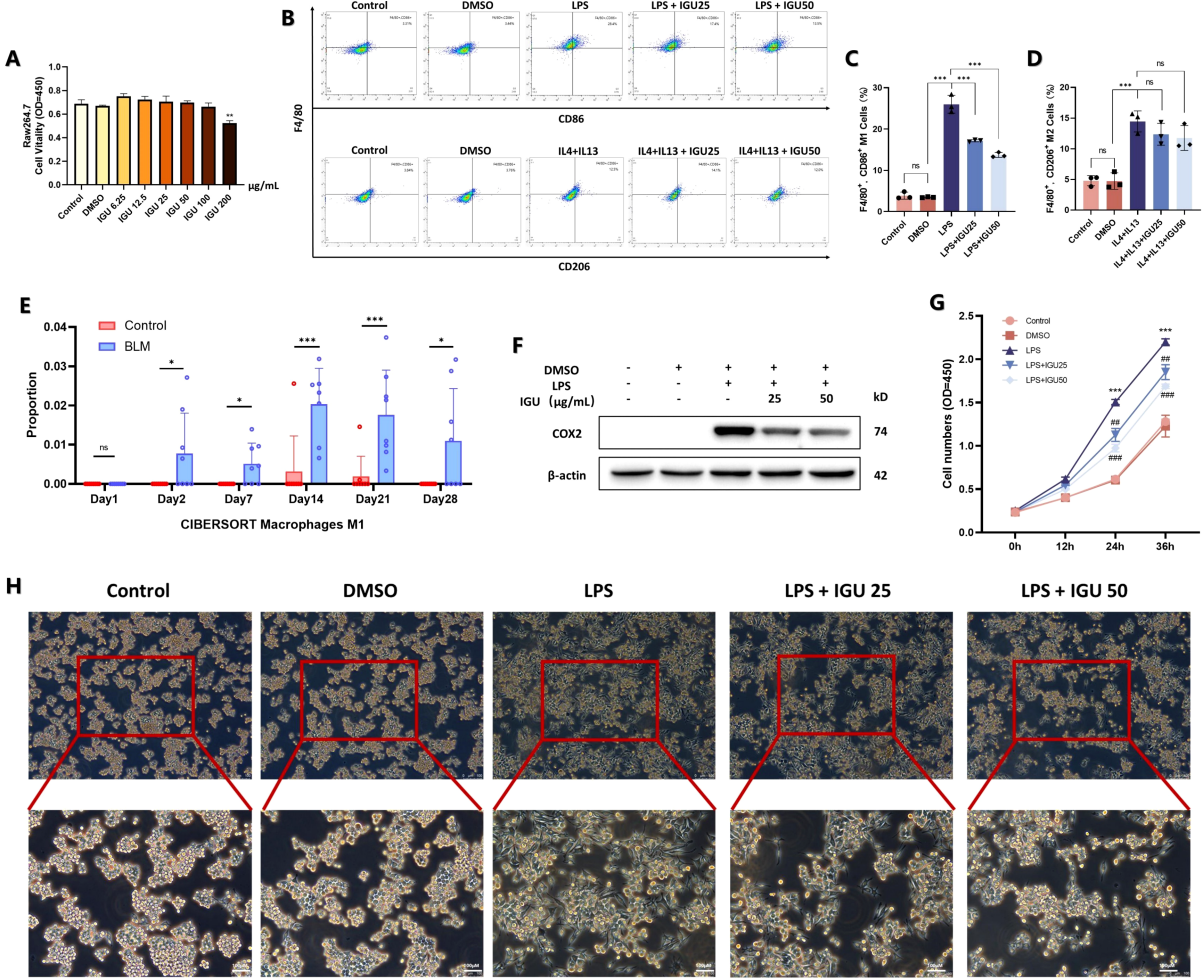
Effect of IGU on M1/M2 polarization of Raw264.7 macrophages. **(A)** CCK8 assay for toxicity of different concentrations of IGU on Raw264.7 cells. **(B–D)** Flow cytometry of the effect of IGU on M1/M2 polarization of Raw264.7 cells (*n*=3). **(E)** CIBERSORT scores for M1 macrophages in the GSE40151 dataset (*n*=8). **(F)** Representative western blot for the protein levels of COX-2 in Raw264.7 cells. β-actin was used as an internal control (*n*=3). **(G)** CCK8 assay for the effect of IGU on the proliferative capacity of M1 macrophage-differentiated Raw264.7 cells (*n*=3). **(H)** Morphological effects of IGU on M1 macrophages under light microscopy. *P<0.05, **P<0.01, ***P<0.001, ns, No significance.

To further investigate the impact of IGU on macrophage polarization, Raw264.7 cells were used to assess the *in vitro* effects of IGU. CCK8 was used to detect the cytotoxicity of IGU on Raw264.7 cells to determine the appropriate concentration. Combined with the reported doses of IGU, 25 μg/mL and 50 μg/mL IGU were used as the treatment concentration for subsequent *in vitro* studies (*p*<0.01, **Figure 5A**). Flow cytometry was used to observe the effect of IGU on different types of macrophages. IGU inhibited the polarization of M1 macrophages, but had no significant effect on M2 macrophages (**Figures 5B–D**). CIBERSORT analysis of the GSE40151 dataset showed the important role of M1 macrophage polarization in PF (*p*<0.05, **Figure 5E**). Consequently, we explored whether IGU could modulate M1 macrophage polarization, as this may underlie its therapeutic effects on PF. IGU, at 25μg/mL and 50μg/mL, inhibited the expression of Cox-2 in LPS-stimulated Raw264.7 cells (*p*<0.001, **Figure 5F**), as well as attenuated the morphological changes (**Figure 5H**) and proliferation (**Figure 5G**).

**Figure 6:**
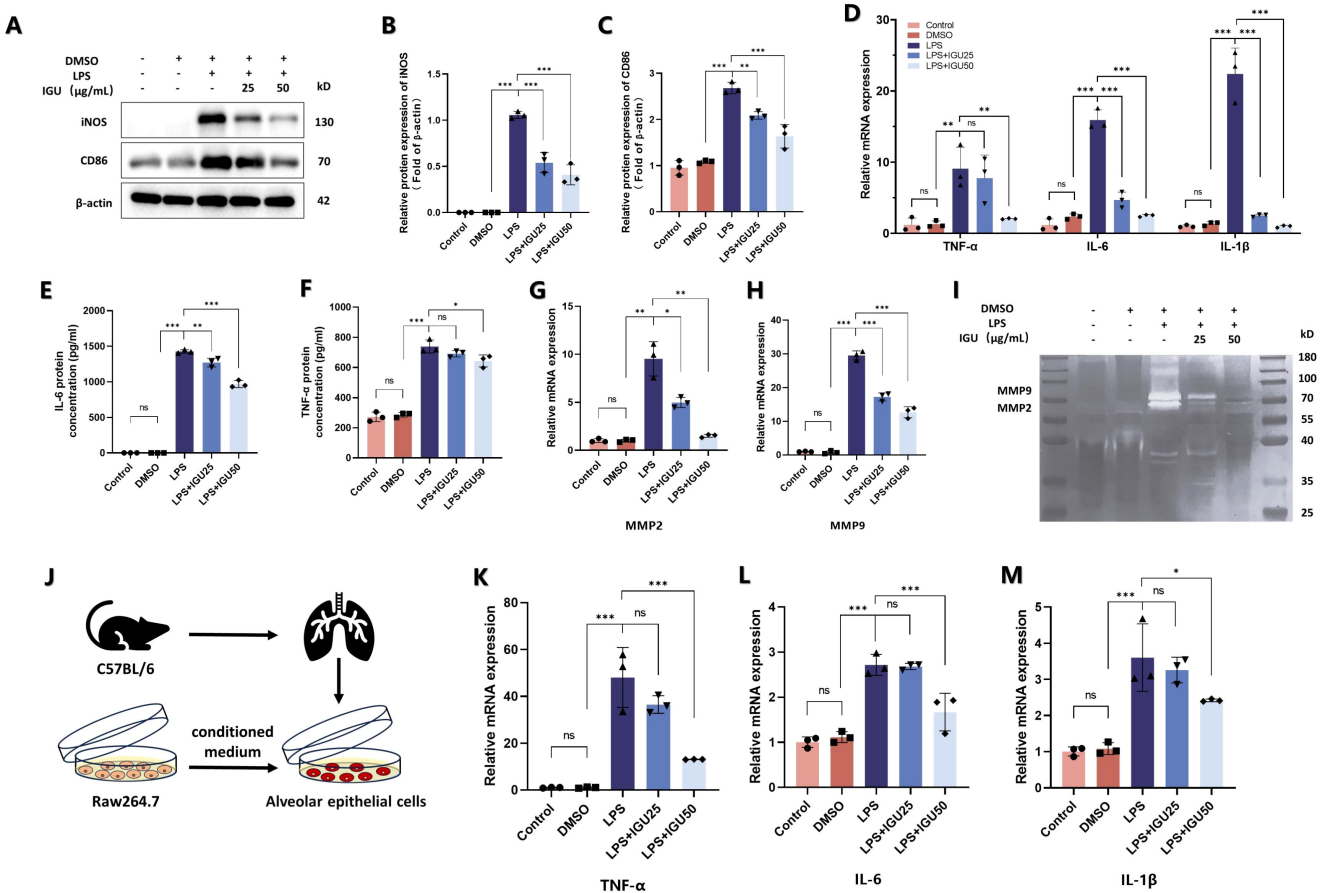
IGU alleviates the inflammation of type II alveolar epithelial cells caused by M1 macrophages. **(A–C)** Representative western blot and quantitation for iNOS and CD86 in Raw264.7 cells. β-actin was used as an internal control. **(D)** QRT-PCR for the mRNA levels of M1 macrophage polarization-related inflammatory cytokines TNF-α, IL-6 and IL-1β in Raw264.7 cells. **(E, F)** ELISA was used to measure the level of TNF-α and IL-6 in Raw264.7 cell culture supernatant. **(G, H)** QRT-PCR for the mRNA levels of MMP2 and MMP9. **(I)** Representative gelatin zymogram of MMPs in Raw264.7 cell culture supernatants. **(J)** Schematic representation of Raw264.7 cells co-cultured with type II alveolar epithelial cells. **(K–M)** QRT-PCR for the mRNA levels of TNF-α, IL-6 and IL-1β in type II alveolar epithelial cells (*n*=3). *P<0.05, **P<0.01, ***P<0.001, ns, No significance.

Subsequently, M1 macrophage-associated markers were examined by western blotting, ELISA and qRT-PCR. Both 25 μg/mL and 50 μg/mL IGU inhibited the increase of CD86 and iNOS protein levels induced by LPS (*p*<0.01, **Figures 6A–C**). In addition, the RNA expression level of inflammatory cytokines, such as TNF-α, IL-1β and IL-6, and the amounts of inflammatory cytokines secreted into the cell supernatant were inhibited by IGU in a concentration-dependent manner (*p*<0.05, **Figures 6D–F**). When macrophages are polarized to M1, they secrete a large amount of matrix metalloproteinases, such as MMP2 and MMP9, which promote the degradation of extracellular matrix and facilitate the infiltration of inflammatory cells into damaged tissues to expand the inflammatory response. The expression levels of MMP2 and MMP9 were detected by qRT-PCR and gelatin zymography, which confirmed the above results (*p*<0.05, **Figures 6G–I**).

Finally, the conditioned medium of each group was collected and added to mouse type II alveolar epithelial cells in culture (**Figure 6J**). The conditioned medium of IGU-treated macrophages reduced the expression of TNF-α, IL-1β and IL-6 in type II alveolar epithelial cells compared with the LPS group (*p*<0.05, **Figures 6K–M**). Thus, IGU inhibited M1 macrophage polarization and ameliorated the inflammation of type II alveolar epithelial cells.

### 3.5 IGU inhibits M1 macrophage polarization through the TLR4/NF-κB signaling pathway

**Figure 7:**
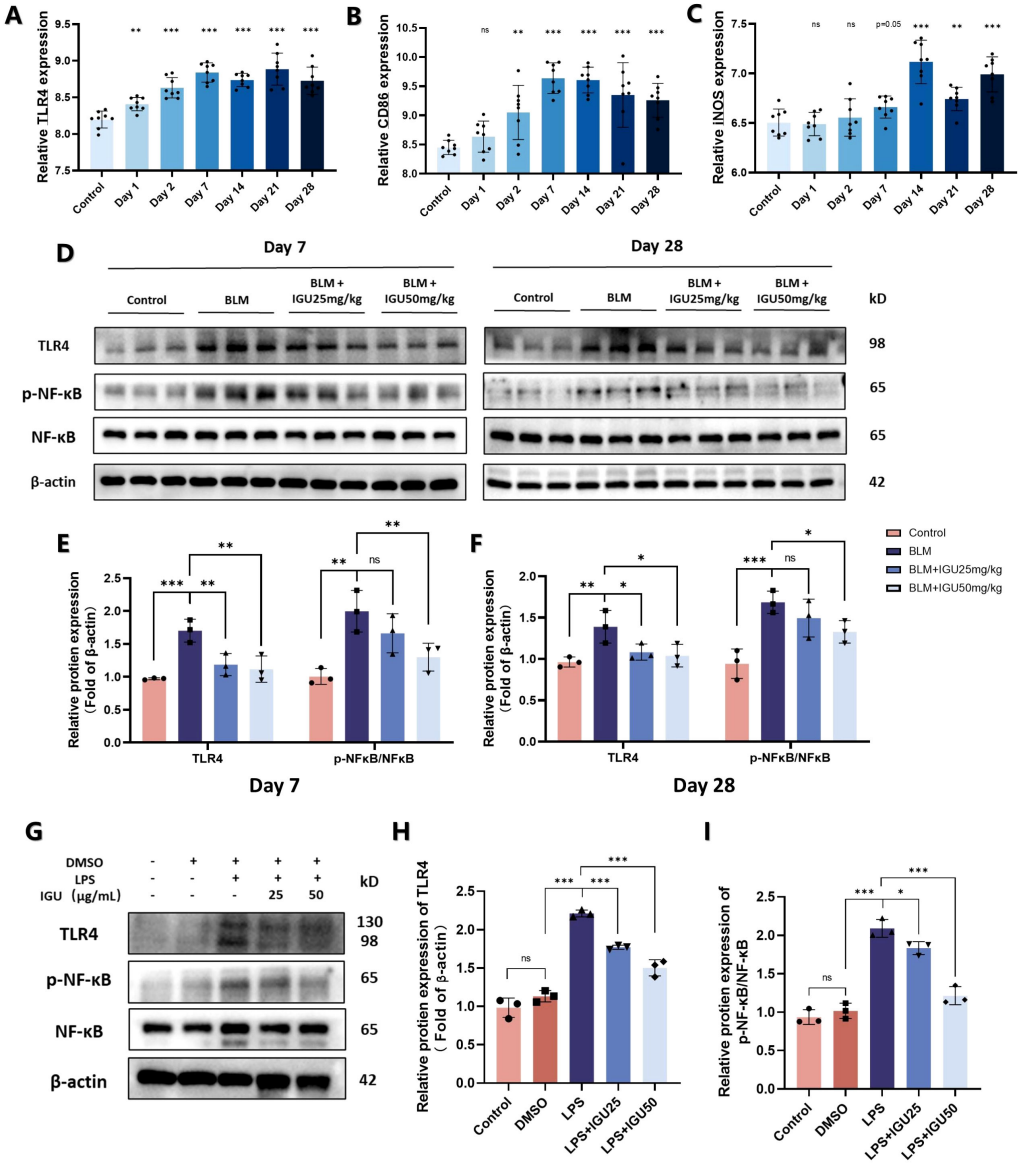
IGU inhibits M1 macrophage polarization through the TLR4/NF-κB signaling pathway. **(A–C)** Expression levels of TLR4, CD86 and iNOS in the GSE40151 of BLM-induced PF in mice (*n*=8). Representative western blots for the protein levels of TLR4 and NF-κB in lung tissues of mice at different stages of BLM-induced PF **(D–F)** and M1-polarized Raw264.7 cells **(G–I)** β-actin was used as an internal control. (n=3). *P<0.05, **P<0.01, ***P<0.001, ns, No significance.

TLR4, a key membrane protein that activates the NF-κB pathway to induce macrophage M1 polarization. The results of the GSE40151 dataset showed that expression of TLR4 in lung tissues was increased at different time points of BLM induction, which was consistent with the expression of M1 macrophage markers CD86 and iNOS (*p*<0.01, **Figures 7A–C**). Therefore, *in vivo* experiments were used for further validation. The protein level of TLR4 was increased and its downstream NF-κB pathway was activated in BLM-induced PF mice on days 7 and 28. However, activation of the TLR4/NF-κB pathway, induced by BLM, was attenuated by IGU treatment (*p*<0.05, **Figures 7D–F**). Similar results were acquired in the Raw264.7 *in vitro* (*p*<0.05, **Figures 7G–I**).

**Figure 8:**
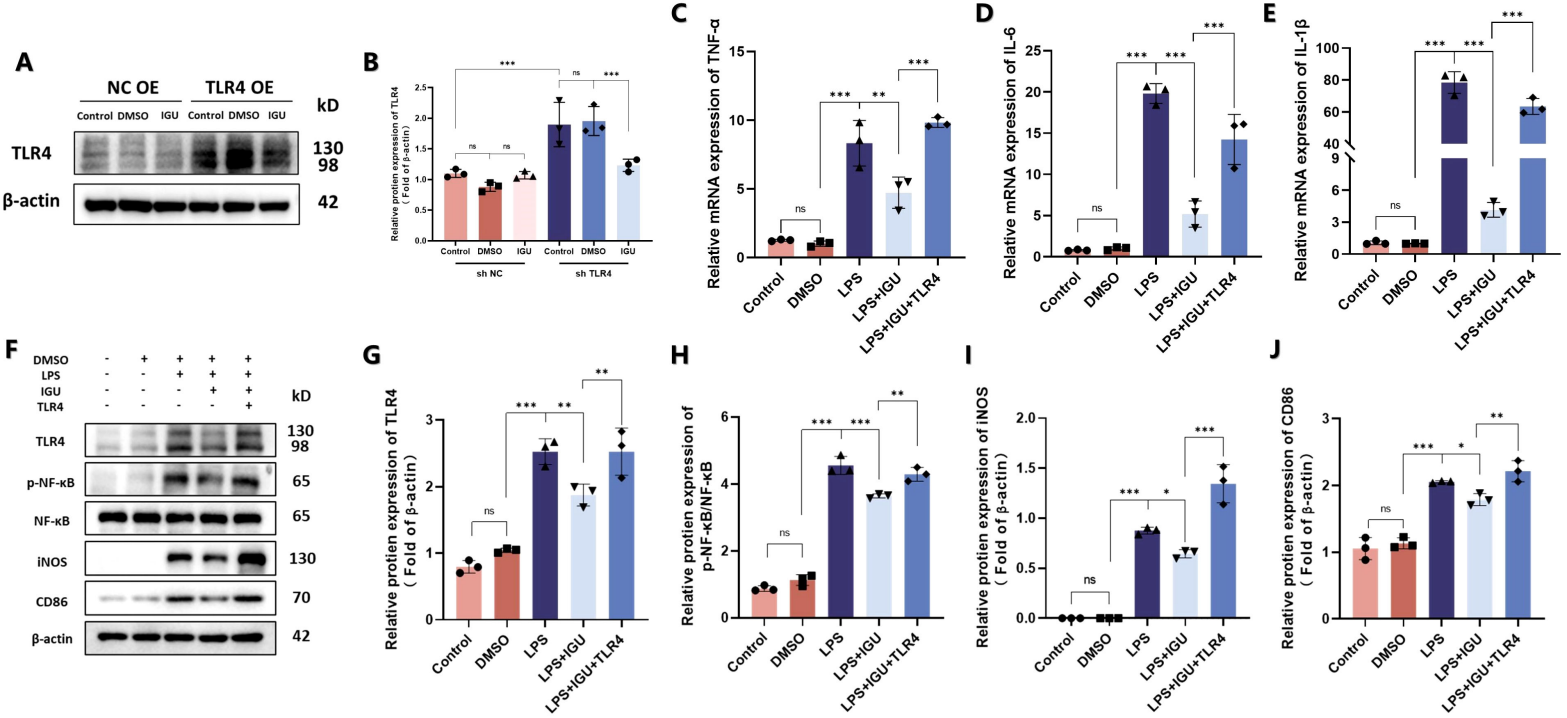
Rescue experiments of Raw264.7 cells after overexpression of TLR4. **(A, B)** Representative western blot and quantitation of TLR4 in TLR4-overexpressing Raw264.7 cells. β-actin was used as an internal control. **(C–E)** QRT-PCR for the mRNA levels of M1 macrophage polarization-related inflammatory cytokines TNF-α, IL-6 and IL-1β in Raw264.7 cells. **(F–J)** Representative western blot and quantitation of protein levels of TLR4, NF-κB, iNOS and CD86 in Raw264.7 cells β-actin was used as an internal control. (n=3). *P<0.05, **P<0.01, ***P<0.001, ns, No significance.

Subsequently, Raw264.7 cells with high TLR4 expression were engineered by lentivirus transduction to construct an *in vitro* rescue experiment. Treatment with IGU resulted in a reduction of TLR4 protein expression in Raw264.7 cells overexpressing TLR4 (*p*<0.001, **Figures 8A, B**). In the TLR4 rescue model, western blotting and qRT-PCR analyses revealed that TLR4 overexpression counteracted the inhibitory effect of IGU on the NF-κB signaling pathway. Correspondingly, the expression levels of the M1 macrophage markers iNOS and CD86, as well as the pro-inflammatory cytokines TNF-α, IL-6, and IL-1β, mirrored this response (*p*<0.05, **Figures 8C–J**). In conclusion, these findings suggest that IGU inhibits M1 macrophage polarization through the TLR4/NF-κB pathway, which may be a significant mechanism by which IGU ameliorates PF.

## 4 Discussion

ILD is a common complication of many rheumatic immune diseases, such as RA, whose treatment is difficult and the effect is not ideal. If ILD involves or develops into PF, the disability and mortality of patients are dramatically increased (25). IGU is a commonly used new disease-modifying anti-rheumatic drug for the management of RA and other rheumatic diseases. Treatment with IGU has been shown to improve key pulmonary function parameters, including forced vital capacity percent predicted, 6-minute walk distance and diffusion capacity for carbon monoxide percent predicted. These findings suggest that IGU not only attenuates systemic inflammation and disease activity but also enhances pulmonary function in patients with RA-ILD, underscoring its dual anti-inflammatory and pulmonary-protective effects (26). In addition, an increasing number of researchers are finding that IGU has anti-organ fibrosis functions, such as countering skin fibrosis in systemic sclerosis (27) and kidney fibrosis in SLE mice (28), as well as anti-pulmonary fibrosis. As in previous studies, we induced pulmonary fibrosis in mice by intratumoral injection of bleomycin (21, 29). After intragastric administration of IGU, we observed the growth and pathology of the mice and found therapeutic effects on lung inflammation on day 7, and on lung fibrosis on day 28. Treatment with IGU showed a lower lung weight index, less pulmonary inflammation and collagen deposition, and less HYP formation in mice. Although the anti-fibrotic effect of IGU was known, the specific mechanism of action was not clear, so the aim of this study was to explore the pharmacological mechanism of IGU in reversing pulmonary fibrosis.

Previous studies have shown that PF is inseparably linked to EMT. Under long-term stimulation of lung inflammation, the body will secrete anti-inflammatory factors, such as TGF-β, to promote tissue regeneration and repair (30). However, the continuous activation of this process will result in excessive tissue repair whereby lung epithelial cells will differentiate into collagen-secreting mesenchymal cells, resulting in lung tissue collagen deposition, and eventually fibrosis (31). In the present study, we found that IGU-treated PF mice had reduced TGF-β secretion increased expression of E-cadherin, down-regulated expression of mesenchymal markers vimentin and α-SMA, and less collagen I deposition. These results indicate that IGU can improve lung tissue fibrosis by preventing EMT, which is consistent with the results of Liu et al (21). We focused on the factors that affect EMT in pulmonary fibrosis, including macrophage polarization. At the same time, we found in the database that in the early stage of BLM-induced PF in mice, lung inflammation was active, and indicators related to M1 macrophages, such as CD86, iNOS, TNF-α, and IL-6, were increased. Long-term inflammatory stimulation leads to excessive activation of TGF-β signaling pathway in the late stage, leading to the formation of PF. These results are also consistent with the disease progression of PF. In the present study, we found that IGU treatment reduces the number of M1 macrophages and the secretion of related pro-inflammatory factors, thereby reducing inflammatory stimuli in the lung, so as to reduce the activation of large number of macrophages and the formation of M2 macrophages in the advanced stage of PF *in vivo*.

More and more studies have shown that macrophage polarization is a continuous process. M1 and M2 macrophages can adjust their polarization according to the changes of the environment, and can transform into each other or depolarize (32, 33). However, there are many influencing factors and the process is complex. Therefore, it is important to pay attention to the dominant population of macrophages during disease progression. Our *in vivo* study shows that IGU inhibits the dominant M1 macrophages in the early stage of pulmonary fibrosis and the dominant M2 macrophages in the late stage of pulmonary fibrosis. To further determine the effect of IGU on the polarization of different types of macrophages, we further validated the effect *in vitro*. The results suggested that IGU had a significant inhibitory effect on the polarization of M1 macrophages, but had no significant effect on the M2 macrophage polarization. IGU inhibits the expression of CD86, the synthesis of iNOS and the secretion of TNF-α, IL-6 and IL-1β in Raw264.7 cells, which is similar to the finding by Hang et al. that treatment with IGU inhibits M1 macrophage formation and thus ameliorates injury after renal transplantation (34). Matrix metalloproteinases (MMPs) are proteases that degrade all components of the extracellular matrix (35). Studies have shown that MMPs have pro-fibrotic effects whose levels are elevated in blood and/or lung samples of pulmonary fibrosis (36, 37). In addition, we note that Zhao et al. showed that one of the important mechanisms by which IGU reduces PF is to inhibit MMP9 expression in lung tissue (38). When stimulated by inflammation, macrophages secrete MMP2 and MMP9 to promote degradation of the surrounding matrix, thereby expanding the inflammatory response, which may be one of the reasons why the expression of MMPs is positively correlated with pulmonary fibrosis (39). Our study found that IGU reduces secretion of MMPs by Raw264.7 macrophages in response to inflammatory stimulation. Combined with other studies, our results also provide a basis for IGU to reduce pulmonary fibrosis.

TLR4, a pattern recognition receptor, is predominantly located on the cell membrane of immune cells, including macrophages, dendritic cells, and monocytes (40). It is well known that TLR4 is a highly specific recognition molecule for LPS. A growing number of studies have shown that TLR4 can also be activated by endogenous molecules, such as glycoproteins, intracellular peptides, and phospholipids, in patients with PF (41). TLR4 deficiency ameliorates BLM-induced PF in mice, suggesting TLR4 activation is closely related to the pathogenesis of pulmonary fibrosis (42). In addition, TLR4 is also a key molecule for inducing the M1 polarization of macrophages (43). External inflammatory stimulation leads to the activation of TLR4, which induces the phosphorylation of NF-κB and the release of IL-1β, IL-6, TNF-α to induce the immune response and inflammation, which may also be an important mechanism by which TLR4 is involved in the pathogenesis of PF (44, 45). In the present study, we found that IGU reduces the expression of TLR4 and its downstream NF-κB pathway in PF mice. Further validation was performed *in vitro* where we found that IGU also reduces LPS-induced activation of the TLR4/NF-κB pathway in macrophages. Thus, M1 macrophage polarization is reduced. TLR4 rescue experiments also showed similar results. Moreover, previous work by Liu et al. demonstrated that IGU also reduces NLRP3 inflammasome expression in PF mice (21). NLRP3 inflammasome formation is closely linked to TLR4/NF-κB pathway activation and M1 macrophage polarization, these findings further substantiate our hypothesis.

**Figure 9:**
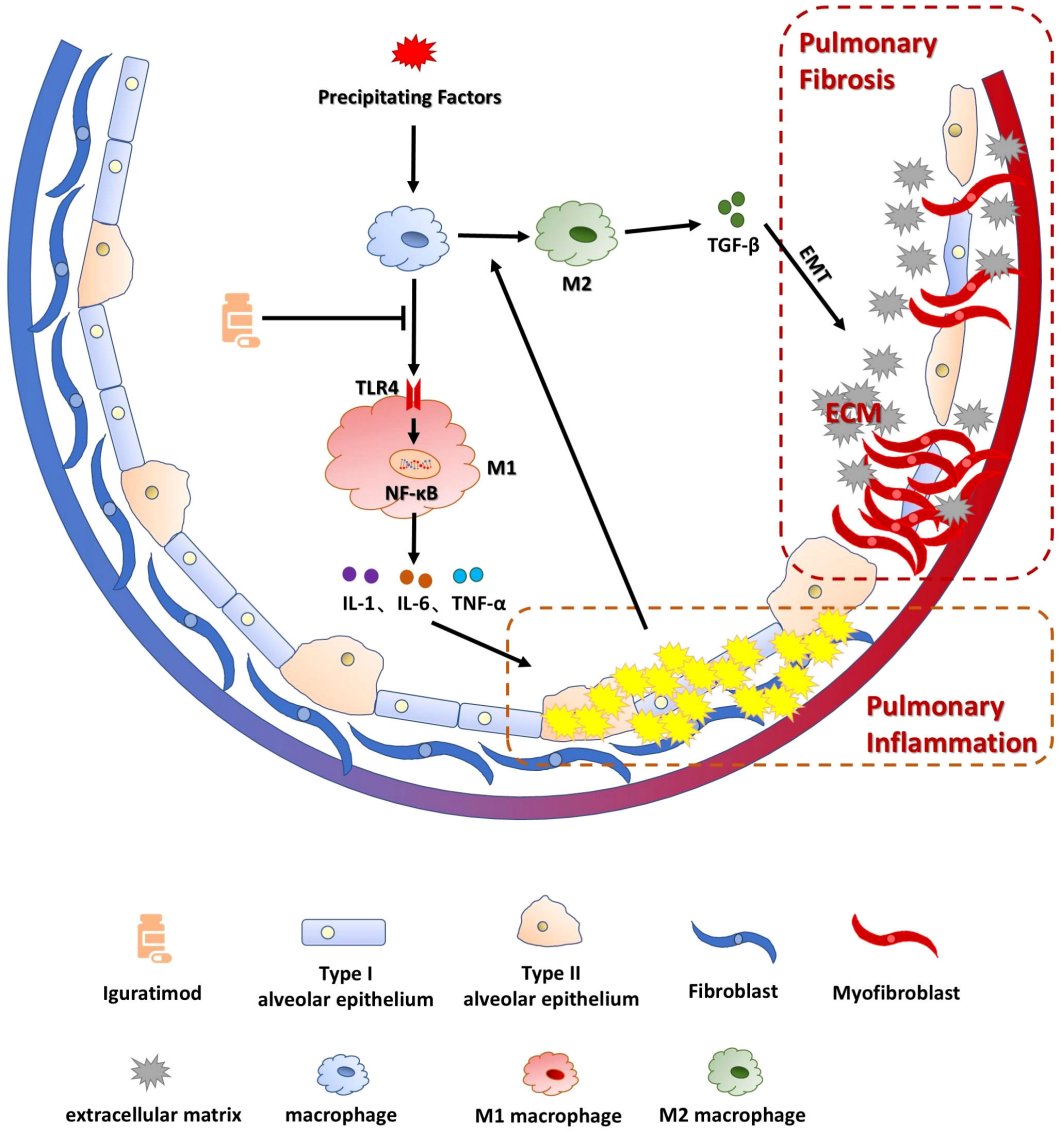
Schematic representation of the mechanism by which IGU ameliorates BLM-induced pulmonary inflammation and fibrosis in mice. IGU ameliorates BLM-induced pulmonary inflammation in mice by blocking the TLR4/NFκB pathway and inhibiting macrophage activation as well as M1 macrophage polarization, thereby reducing PF caused by the polarization of M2 macrophages and EMT in the advanced stage of the disease.

In conclusion, IGU ameliorates BLM-induced pulmonary inflammation in mice by blocking the TLR4/NFκB pathway, suppressing macrophage activation, and preventing M1 macrophage polarization, thereby reducing PF caused by the polarization of M2 macrophages and EMT in the advanced stage (**Figure 9**). However, there are some limitations in this study. Clinical validation and translation are the difficulties of this study. Evidence suggests that CTD patients treated with IGU have better therapeutic efficacy and lower incidence of interstitial lung disease (46), suggesting that IGU may become a new treatment option for interstitial pulmonary fibrosis. Clinical validation would be the next step in this study, such as the incidence of PF in RA patients after taking iguratimod, and the improvement of RA-ILD patients or PF patients after taking IGU, so as to achieve the purpose of clinical transformation.

## Ethics approval and consent to participate

The experiment was mainly completed in the Laboratory of Molecular Cardiology, First Affiliated Hospital of Shantou University Medical College. All experimental procedures *in vivo* were approved by the Animal Ethics Committee of Shantou University Medical College (approval number SUMC2024-001).

## Data Availability

The datasets presented in this study can be found in online repositories. The names of the repository/repositories and accession number(s) can be found in the article/supplementary material.
